# Scrotal Pyoderma Gangrenosum Associated with Evans Syndrome

**DOI:** 10.3390/jcm7090230

**Published:** 2018-08-22

**Authors:** Deng-Ho Yang, Meng-Yin Yang

**Affiliations:** 1 Division of Rheumatology/Immunology/Allergy, Department of Internal Medicine, Taichung Armed-Forces General Hospital, Taichung 411, Taiwan; 2 Department of Laboratory, Taichung Armed Forces General Hospital, Taichung 411, Taiwan; 3 Department of Medical Laboratory Science and Biotechnology, Central Taiwan University of Science and Technology, Taichung 406, Taiwan; 4 Division of Rheumatology/Immunology/Allergy, Department of Internal Medicine, Tri-Service General Hospital, National Defense Medical Center, Taipei 114, Taiwan; 5 Department of Neurosurgery, Jan-Ai General Hospital, Taichung 412, Taiwan; yangmy04@gmail.com; 6 Department of Neurosurgery, Tri-Service General Hospital, National Defense Medical Center, Taipei 114, Taiwan; 7 College of Nursing, Central Taiwan University of Science and Technology, Taichung 406, Taiwan; 8 Department of Neurosurgery, Taichung Veterans General Hospital, Taichung 407, Taiwan

**Keywords:** pyoderma gangrenosum, Evans syndrome, ulcer, steroid

## Abstract

Evans syndrome is a rare disorder with presentations of autoimmune hemolytic anemia and immune thrombocytopenia, in the absence of any underlying cause. Here, we reported a case with a history of Evans syndrome for seven years. A persistent scrotal ulcer with severe pain occurred for two weeks. He called at our emergency room because of a painful, necrolytic cutaneous ulcer over the scrotal region. A biopsy showed sterile dermal neutrophilia with lymphocytic vasculitis, and pyoderma gangrenosum was impressed. The patient received steroid treatment and recovery after one month.

## 1. Introduction

Pyoderma gangrenosum (PG) is an uncommon dermatologic finding with equal gender distribution, and is often misdiagnosed as an infection. Half of the patients with PG are associated with underlying systemic diseases, including inflammatory bowel disease, hematologic malignancy, paraproteinemia, and Behcet’s disease [1,2]. The most common underlying systemic diseases associated with PG are inflammatory bowel disease [3]. Initially, some patients with PG are found to have been misdiagnosed. The diagnosis is based on a history of rapidly progressive disease course, and underlying disease and histologic finding. The different subtypes of PG include ulcerative, bullous, pustular, and vegetative manifestations [1]. Most of the patients with PG have a rapid response to systemic steroid treatment. PG in the patients with Evans syndrome is rare. We presented a case of scrotal PG associated with Evans syndrome, and the site of occurrence is uncommon. This is the first case report about PG occurring in a patient with Evans syndrome.

## 2. Case Report

**Figure 1:**
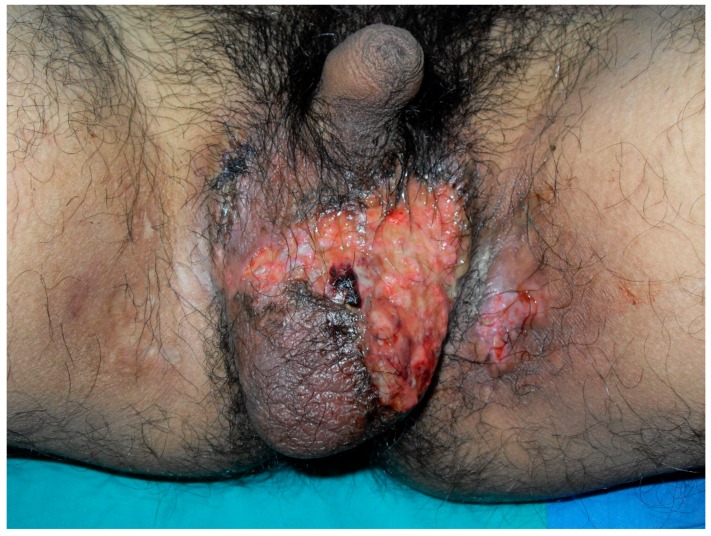
A painful destructive ulcer with an irregular margin and erythematous edges on the anterior scrotal wall.

**Figure 2:**
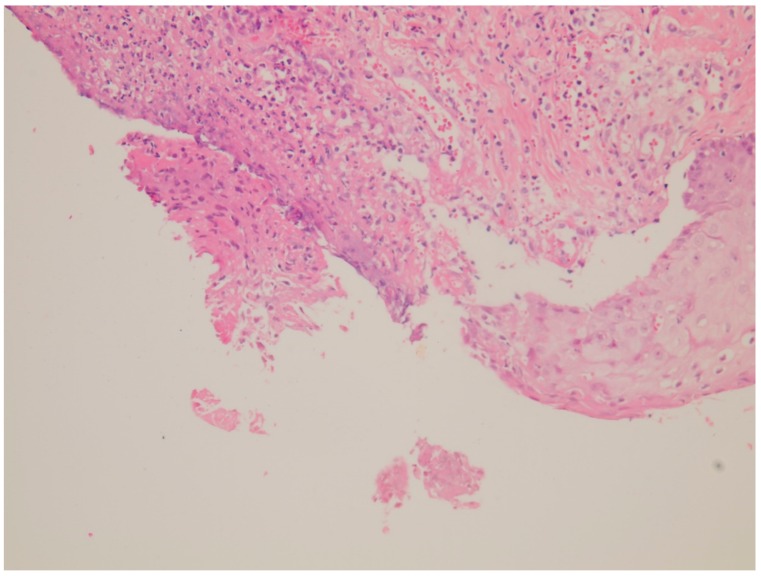
Ulcer with necrotizing inflammation and abcess formation over the dermis (hematoxylin and eosin stain, 200×).

**Figure 3:**
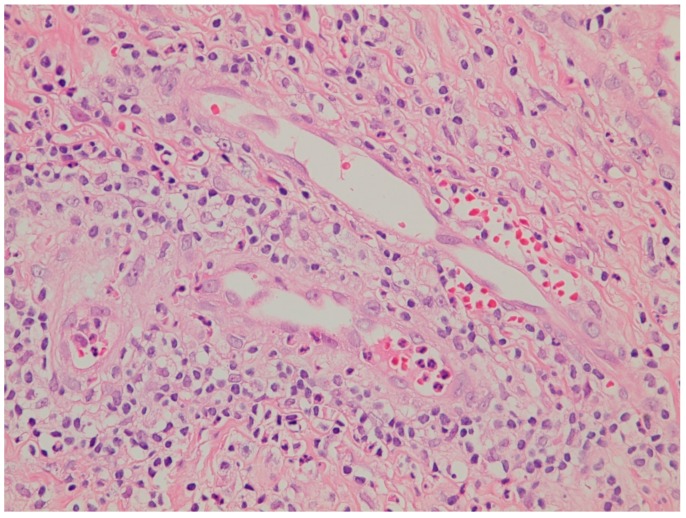
Dermal neurophilic infiltration with mixed inflammation and focal lymphocytic thrombogenic vaculopathy (hematoxylin and eosin stain, 500×).

**Figure 4:**
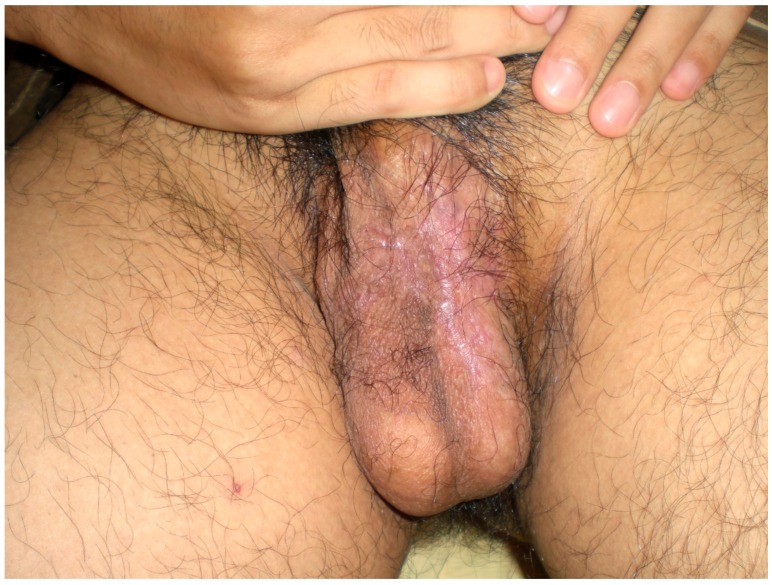
The scrotal ulcer of pyoderma gangrenosum (PG) was totally healed after one month.

A 33-year-old male presented with a painful scrotal ulcer of two weeks’s duration. Initially, an ulcerative papule occurred over scrotum and enlarged rapidly. He had a history of Evans syndrome diagnosed by autoimmune hemolytic anemia (AIHA) and immune thrombocytopenia (ITP), since seven years ago. A bone marrow study showed a hypercellular pattern without malignancy. The long time medication of predinsolone was given to control the AIHA and ITP. An intermittent blood transfusion with blood and platelets was also performed during a recurrent acute episode, with a poor control of anemia and thrombocytopenia. Six months before this admission, the patient discontinued medication of steroids by himself. On examination, a 5-cm painful destructive ulcer with an irregular margin and erythematous edges on the anterior scrotal wall was found (Figure 1). He did not have any joint or eye complaints and has never had sexual intercourse. The skin pathergy test was negative. No other skin or muscosal ulcer was found. Laboratory data showed a white blood cell count of 8500/mm^3^, platelet count of 36,000/mm^3^, hemoglobin of 7.3 g/dL, and serum C-reactive protein of 9.5 mg/dL. The features of hemolysis, including a low hepatoglobin level, elevated lactate dehydrogenase, bilirubin levels, and direct antiglobulin test, were positive. Rheumatoid factor, anti-neutrophilic cytoplasmic antibodies, anti-cardiolipin antibody, anti-dsDNA, and anti-nuclear antibody were negative. No monoclonal gammopathy was detected on the protein electrophoresis. The microbiological tissue cultures for bacteria, fungi, and mycobacteria were negative. A biopsy of the scrotal ulcer was done and showed the ulcer with necrotizing inflammation, with a focal lymphocytic thrombogenic vasculopathy (Figure 2 and Figure 3). PG was impressed and the patient received intravenous methylprednisolone 120 mg daily for four days. The scrotal ulcer was improved progressively, and the C-reactive protein was decreased (1.1 mg/dL) after one week. He was then given the medication including prednisolone 20 mg twice daily and azathioprine 50 mg daily. After two weeks of immunotherapy, a significant reepithelialization of the ulcer was found. One month after the initial presentation, the scrotal ulcer of PG was totally healed (Figure 4).

## 3. Discussion

Evans syndrome is an autoimmune disorder characterized by the development of AIHA and ITP, and/or immune neutropenia, in the absence of any underlying cause. Evans syndrome is a disorder of immune regulation, with unknown phathophysiology. Dysregulation and abnormalities in cellular and humoral immunity can be found in Evans syndrome. Clinical presentations of Evans syndrome include the usual features of hemolytic anemia (jaundice, pallor, and dizziness) and thrombocytopenia (petechiae and mucocutaneous bleeding) [4]. There are many secondary causes of Evans syndrome, including systemic lupus erythematosus, primary antiphospholipid syndrome, Sjogren’s syndrome, primary immunodeficiency, lymphoma, leukemia, and lymphoproliferative disorders [5,6,7]. Thus, the diagnosis of Evans syndrome still needs to exclude underlying diseases or conditions that may influence the management of the prognosis. In our patient, we could not find other underlying diseases or conditions causing Evans syndrome, as a result of negative autoantibodies (including anti-nuclear antibody, anti-dsDNA antibodies, and anticardiolipid antibodies), a negative human immunodeficiency virus test, and no malignancy from a bone marrow biopsy. 

PG is an unusual inflammatory skin disease that typically begins as nodules or sterile pustules that rapidly evolve into painful cutaneous ulceration. It is difficult to diagnose and is always associated with numerous systemic diseases initially. Careful history taking and clinical pathologic findings are necessary to make an accurate diagnosis. The diagnosis of PG is a diagnosis of exclusion. The differential diagnosis for cutaneous ulceration includes ischemic or neurotropic venous diseases and nonvascular diseases. Most of the misdiagnosis of PG includes vascular occlusive or venous diseases, venous stasis ulceration, livedoid vasculopathy, and vasculitis. PG can occur in different body sites, including legs, trunk, face, intestine, and pulmonary [1]. However, genital PG with the involvement of the vulva, penis, or scrotum is rare [8,9,10,11,12,13,14,15]. The systemic diseases may be associated with the development of genital PG (Table 1). In the patient with PG, an excellent improvement of the cutaneous ulcer can be found after adequate steroid medication. In ulcerative PG, the histopathology shows sterile dermal neurophilia with mixed inflammation and lymphocytic vasculitis. Therefore, ulcerative PG was favored in our patient. PG is usually associated with autoimmune or hematological diseases, including inflammatory bowel disease, rheumatoid arthritis, hepatitis C, leukemia, myelodysplasia, and monoclonal gammopathies [16]. Among these disorders, different types of autoantibodies can be found in clinical. Although the mechanisms underlying PG are not fully understood, a good response to immunomodulatory drugs such as corticosteroids, Tumor necrosis factor-α inhibitor, and calcineurin inhibitor, supports an immune-mediated mechanism of PG [8,17,18]. Evans syndrome is a systemic disease of immune dysregulation with circulation autoantibodies to red cells or platelets. Adequate immunosuppresion by corticosteroids, intravenous immunoglobulin, calcineurin inhibitor, and rituximab was recommended during the remission or exacerbation of Evans syndrome [19,20]. PG and Evans syndrome are both immune mediated systemic diseases. PG can be worsening or expedited during the interval of the patients receiving immunosuppressive therapy. Evans syndrome and PG are both autoimmune diseases. PG is associated with active soft tissue inflammation by infiltration of neutrophils. Evans syndrome is associated with autoimmune cytopenias by alterations of immune regulation. Abnormal immune regulation may induce aggressive autoimmune inflammation [13]. Our patient had a history of Evans syndrome. He never received aggressive immunosuppressive therapy and stopped his medication for six months. The discontinuation of therapy may result in a PG flare. 

Initial immunosuppressive management should be given, and systemic corticosteroids are usually suggested (1–2 mg/kg/day). Other potential drugs, including cyclosporine, azathioprine, methotrexate, colchicine, and TNF-α inhibitor, can be employed for refractory PG [1]. Topical corticosteroids or immunomodulators can be directly used on the ulcers, and surgical intervention or debridements should be avoided. An aggressive evaluation of the underling disease, such inflammatory bowel disease or hematologic disorders, is still important. Control of the underlying diseases and treatment of the PG itself should both be performed.

## 4. Conclusions

In summary, PG is a recurrent cutaneous ulceration and is usually associated with underlying diseases. Evans syndrome may be one of the possible underlying causes. Our case illustrates the rare presentation of scrotal PG. Among patients with refractory ulcerations without evidence of infection, the diagnosis of PG should be considered. An early diagnosis of PG by clinical features and histophathologic findings is important, because of a good response from systemic corticosteroid medication.

## Figures and Tables

**Table 1:** The systemic diseases associated with genital pyoderma gangrenosum (PG).

Autoimmune diseases	Crohn’s disease
	Ulcerative colitis
	Behcet’s disease
	Dermatomyositis.
	LPIN2 gene mutation related autoinflammatory syndrome
Hematologic diseases	Myelodysplastic syndrome
	Evans syndrome
Malignancy	Lymphoma
	Acute myeloid leukemia
	Cutaneous squamous cell carcinoma
	Prostate cancer
Infection	human immunodeficiency virus
	Hepatitis C infection
	Tuberculosis
Drugs	Rituximab
	Infliximab
